# Provincial Medical and Surgical Association

**Published:** 1836-07

**Authors:** 


PROVINCIAL MEDICAL AND SURGICAL ASSOCIATION.

We much regret that the fourth volume of the Transactions of the Provincial Medical and Surgical Association has reached us too late for review. The volume extends to nearly 600 pages, and is enriched by the address delivered at Oxford, in July last, by Dr. Prichard, and by many valuable papers. We are glad to observe that it is rich in medical topography. The volume is opportunely published: the fourth anniversary meeting of the Association is to be held at Manchester, at the rooms of the Royal Institution, in Mosley street, on Wednesday the 20th, and Thursday the 21st of July next; and we are glad to see that the Council have taken much pains to inform the members beforehand what will be the order of business at the meeting. The president, Edward Holme, M.D., will deliver his address at seven o'clock on Wednesday evening, at the theatre of the Royal Institution; after which, the Report of the Council will be read. The Reports of the Committees appointed at the meeting at Oxford will then be received; and, if time will permit, will be read. On Thursday morning, at twelve o'clock, the members will again assemble at the theatre of the Royal Institution, and the Retrospective Address will be delivered by John Green Crosse, Esq. F.R.S., Surgeon to the Norfolk and Norwich Hospital; and the general business of the Association will be transacted. Separate rooms will be appointed for the committee on the Poor-Law Amendment Bill, and the committee of the Benevolent Society; and any gentleman wishing to make any communications on those subjects will be good enough to enquire for the respective committees. Each member, on arriving in Manchester, is directed to proceed to the Royal Institution in Mosley street, where persons will be placed to receive the names and abodes of members as they arrive, and to give every information as to the progress of business. On Thursday, at half-past five o'clock, the members will dine together; and we are particularly glad to learn that, at nine o'clock in the evening of Thursday, the members and their friends will hold a Conversazione, and every arrangement will be made with a view to promote friendly intercourse, and to facilitate the communication between members from different parts of the kingdom. The interest attached to the meeting will doubtless be increased by the arrangements of the Manchester committees. In the morning of Wednesday and Thursday, previously to the meetings of the Association, the rooms of the Natural History Society, the Royal Infirmary and Fever Wards, and some of the manufactories, will be open to the inspection of the members. It is unnecessary to speak of the advantages arising from these meetings; and we have no doubt that the meeting at Manchester will still further increase the influence and the utility of the Provincial Association.

---

EASTERN PROVINCIAL MEDICAL AND SURGICAL ASSOCIATION.

